# FEASIBILITY OF ONLINE SYNCHRONOUS CAREGIVER DEMENTIA COACHING FOR REJECTION-OF-CARE BEHAVIORS

**DOI:** 10.1093/geroni/igz038.3367

**Published:** 2019-11-08

**Authors:** Rita A Jablonski, Winstead Vicki, Giovanna Pilonieta, David Geldmacher

**Affiliations:** University of Alabama at Birmingham, Birmingham, Alabama, United States

## Abstract

Problem: Two-thirds of family caregivers of persons living with dementia have encountered rejection-of-care behavior, usually during assistance with activities of daily living. Purpose: To describe the feasibility of an online videoconferencing platform to help caregivers prevent and reduce ROC behavior. Design: Quasi-experimental. Sample: Twenty-six family caregivers: 54% female, 77% white, 62% spouses (31% wives, 31% husbands), mean age 65 years, and college-educated (92%). Their care recipients were 61% female, 77% white, mean age of 76 years, and college-educated (88%). Procedure: Family caregivers who endorsed problematic ROC behaviors in their care recipients participated in six online, individual, synchronous, sequential, and weekly 1-hour coaching sessions. We measured general burden (Zarit Burden Inventory) and the frequency, severity, and associated distress of responsive behaviors (Neuropsychiatric Inventory Questionnaire). Data collection intervals were before coaching (baseline), immediately after the final session (Time 1), and six weeks (Time 2) and 12 weeks (Time 3) after the final session, respectively. Results: Caregivers reported less overall distress scores at Time 2 compared to baseline: 13.58 (SD 6.44) versus 17.42 (SD 6.90), t=2.56, p=0.017). Distress scores returned to baseline by Time 3. Caregivers reported less severe ROC behavior at Time 2 which was not statistically significant. Burden remained unchanged throughout the 24 weeks. Conclusion: Online caregiver coaching that targets ROC behavior is feasible. Qualitative review of the encounters suggests that a longer period of intervention and an outcome measure more sensitive to ROC effects on activities of daily living may be needed in future studies.

